# Expression of MHC I Isoforms in Bovine Placentomes: Impact of Cloning

**DOI:** 10.3390/vetsci12030196

**Published:** 2025-02-21

**Authors:** Rodrigo da Silva Nunes Barreto, Ana Carolina Furlanetto Mançanares, Maria Angelica Miglino, Flávio Vieira Meirelles, Lilian de Jesus Oliveira

**Affiliations:** 1 RB Lab, College of Agricultural and Veterinary Sciences, Sao Paulo State University, Jaboticabal 14884-900, SP, Brazil; rodrigo.barreto@unesp.br; 2 Department of Animal Science, Faculty of Veterinary Sciences, Universidad de Concepción, Chillán 3780000, Chile; 3 Department of Veterinary Medicine, University of Marília (UNIMAR), Marília 17525-902, SP, Brazil; 4 Department of Veterinary Medicine, Faculty of Animal Sciences and Food Engineering, University of São Paulo, Pirassununga 13635-900, SP, Brazil; 5 Department of Pathology, College of Veterinary Medicine, University of Georgia, Athens, GA 30602, USA

**Keywords:** BoLA, bovine placenta, chorionic villi, cloned bovine, IL-A88, Qa-2

## Abstract

*Major histocompatibility complex class I* (*MHC-I*) gene expression in the placenta is modulated to tailor the maternal immune response to fetal antigens during pregnancy. This study evaluated MHC-I expression through immunohistochemistry (IHC) using an anti-mouse preimplantation embryo development (PED) clone Qa-2 and anti-bovine leukocyte antigen I (BoLA) monoclonal antibody clone IL-A88 (*n* = 23), as well as RT-qPCR (*n* = 17) for classical and non-classical (*BoLA-NC*) genes in control and cloned bovine placentomes during early and near-term gestation. Control samples showed minimal Qa-2 protein expression in early gestation, with intense labeling in trophoblasts and the maternal uterine epithelium near term. In contrast, cloned samples exhibited intense Qa-2 labeling in both maternal and trophoblastic epithelia at both stages, while trophoblast giant cells (TGCs), located apposed to the maternal epithelium, showed no labeling. Control samples exhibited intense IL-A88 labeling in the maternal epithelium at both stages. In cloned samples, weak to no labeling was observed in early gestation, with intense labeling in maternal and fetal epithelium near term. RT-qPCR revealed significant upregulation of *BoLA-NC3* in early gestation, with sustained elevated expression in cloned samples in the near term. These findings suggest that altered BoLA protein expression and gene regulation in cloned pregnancies may contribute to pregnancy complications and increased losses.

## Simple Summary

The immune system protects the body from infections and distinguishes between self and foreign components. During pregnancy, the mother’s immune system recognizes and tolerates the foreign paternal proteins present in the fetus. When there is a maladjustment of the maternal immune system during pregnancy, it may result in maternal rejection and pregnancy loss. The fetus also helps regulate the maternal immune system by producing placenta-specific proteins called non-classical major histocompatibility complexes (MHC-Ib). This study examined how MHC-Ib proteins differ between non-cloned (control) and cloned cow pregnancies since cloned pregnancies have more complications and losses. Using tissue staining and gene expression analyses, we found that in control pregnancies, early expression of a specific MHC-Ib (Qa-2) was barely present but increased near calving, whereas Qa-2 had an opposite pattern in clones. Another MHC-Ib protein (ILA-88) was strongly present throughout the control pregnancies and weaker in early cloned pregnancies, with staining increasing near calving. Additionally, a gene linked to MHC-Ib (BoLA-NC3) showed an opposite expression pattern in cloned pregnancies compared to controls. These changes in MHC-Ib protein levels and gene activity suggest that clones may struggle with proper immune regulation, contributing to the higher rates of pregnancy loss and complications.

## 1. Introduction

During pregnancy, the communication between mother and fetus occurs through the expression and transfer of placental-specific factors [1], such as the fetal expression of major histocompatibility complex (MHC) proteins [2]. In hemochorial placental models, such as in mice and humans, semi-allogeneic trophoblastic cells (fetal cells) decrease the expression of MHC-I molecules early in gestation to avoid the recognition of paternal antigens by the maternal immune system [3]. With the low to no expression of MHC-I, the fetal cells may become a target of uterine natural killer (uNK) cells [4]. To prevent activation of uNK cells, the trophoblast cells express a unique form of MHC molecules: The non-classical (NC) MHC-I isoforms [5], described as *human leukocyte antigen G* (*HLA-G*) or murine *preimplantation embryo development* (*PED*) genes [2]. NC *MHC-I* genes are expressed in isoforms: membrane-bound or soluble proteins [6]. Soluble HLA-G is detected in the maternal blood in successful pregnancies, suggesting a systemic modulation of the maternal immune system [7]. High expression of HLA-G is associated with an invasive phenotype of the trophoblast cells in vitro, the extravillous trophoblasts (EVTs) [8]. However, trophoblast invasion and adhesion to the decidua are regulated not only by HLA-G expression but also through interactions with inhibitory receptors on NK cells and macrophages, such as KIR2DL4/CD158d, LILRB1/2, and ILT2/4 [8,9]. HLA-G expression by the EVT modulates the local maternal immune system through the uNK cell system and its interactions with macrophages and other immune cells. Regulatory T (Treg) cells play an essential role in supporting the semi-allogeneic fetus in successful pregnancies [10], inhibiting T cell-mediated lysis of trophoblastic cells, and promoting a supporting immune environment [11]. Abnormal expression of HLA-G appears deleterious: its downregulation correlates with reduced numbers of CD56^bright^CD16^−^ uNK cells in the maternofetal interface, which can contribute to miscarriages [12]. An abnormal invasion of the EVTs and uNK cells can impair the development and remodeling of maternal spiral arteries, predisposing intrauterine to restrict growth and pre-eclampsia [13]. For example, when the EVTs from mid-gestation samples are cultured in conditioned uNK cell media, their ability to invade is significantly increased in vitro [14]. Moreover, the co-culture of the HLAG^+^ EVTs and CD4^+^ cells increases the expression of forkhead box P3 (FOXP3) by CD4^+^CD25^high^ T reg cells, supporting the HLA-G effects on the Th immune response at the maternofetal interface [10].

In contrast to humans and mice, bovines display a less invasive or epitheliochorial type of placentation in which all maternal tissue layers are maintained throughout pregnancy [14,15]. Unlike other species with epitheliochorial placenta, the ruminant placenta is unique and appears more permeable to fetal products [16]. In cows, the trophoblastic giant cells (TGCs) migrate toward the maternal epithelium and form a hybrid syncytium with the maternal epithelium [16] to deliver fetal products to the maternal system [17]. Despite maintaining all tissue layers in both the dam and fetus sides, the dam immune system displays similar local and systemic modulation as observed in humans and mice. Such include increased circulating CD4^+^CD25^high^ T reg cells in early pregnancy [18] and alternative activation of uterine macrophages near-term [19] The more intimate maternofetal communication in cows primes the maternal system to semi-allogeneic fetal antigens [20]. In contrast to humans and mice, MHC-I or bovine leukocyte antigen class I (BoLA-I) expression in early gestation is unknown. In cows, trophoblasts in the arcade zones and interplacentomal chorionic regions express BoLA-I around 6 months into the pregnancy; meanwhile, BoLA-I expression is absent deep in the chorionic villi [21,22,23,24]. This suggests that the suppression of BoLA-I expression in bovine placentomes occurs in areas of close contact between fetal cells and the maternal epithelium [21,22,23,25]. In clones, BoLA-I is expressed as early as day 36 in the chorioallantois membrane, which later differentiates into chorionic villi as the placentome development progresses [25]. This suggests that BoLA-I expression in early pregnancy may represent a non-classical isoform of BoLA class I (BoLA-NC), like murine PED and human HLA-G [26]. Furthermore, atemporal and increased expression of BoLA-I observed in cloned pregnancies may have contributed to abnormal pregnancy/placentation and losses in the early stages of pregnancy [8,27].

The expression patterns of classical and non-classical isoforms of BoLA class I molecules remain uncharacterized, and their role in local maternal–fetal immune modulation remains unclear. In this study, we describe the expression of classical and non-classical MHC class I molecules in bovine placentomes from both cloned and naturally conceived pregnancies, examined during early and near-term stages of gestation.

## 2. Materials and Methods

For immunohistochemistry (IHC), mouse Qa-2 (clone 69H1-9-9, #11-5996; eBioscience, San Diego, CA, USA) and mouse bovine monomorphic MHC (clone IL-A88, #MCA2444GA, AbDSerotec, Kidlington, Oxford, UK) were used as primary antibodies. For control, we used anti-mouse IgG (clone MOPC-21, #M5284; Sigma-Aldrich, Saint Luis, MO, USA). Reactions were tagged with Dako Advance tm HRP (#K4069) and stained using 3,3-diaminobenzidine (DAB, Liquid DAB+ Substrate Chromogen System, #K346811), both purchased from DAKO (Carpinteria, CA, USA).

For real-time quantitative PCR (RT-qPCR), an RNeasy Plus Mini kit (#74136, Qiagen—Valencia, CA, USA) was employed for RNA extraction, DNAse I amplification grade (#18068-015), alongside RNAseOut Recombinant Ribonuclease Inhibitor (#10777-019), High-Capacity cDNA Reverse Transcription kit with RNase Inhibitor (#4374967), and SyBr Green PCR master mix (#4344463), all purchased from Invitrogen (Foster City, CA, USA). Primers were purchased from Exxtend (Paulínia, SP, Brazil).

Monohydrate citric acid, sodium citrate tribasic dehydrate, sodium chloride (NaCL), hydrogen peroxide (H_2_O_2_), potassium chloride (KCl), Trizma base (C4H11NO3), and 3-(Aminopropyl) triethoxysilane for buffer solutions were purchased from Sigma-Aldrich.

### 2.1. Clustering of BoLA Isoforms and Sequences Recognized by Qa-2 and IL-A88 Antibodies

Published classical and non-classical BoLA isoform protein sequences were retrieved from the IPD-MHC database (https://www.ebi.ac.uk/ipd/mhc/allele/list/?query=eq(organism.group,BoLA)#panelAdvanced, accessed on 8 January 2025) and peptide sequence recognized by the anti-Qa-2 (GenBank: X80933.1) and anti-IL-A88 (GenBank: NM_001201460.1) antibodies were retrieved in FASTA format for further analysis. Phylogenetic analyses were conducted using Clustal Omega (https://www.ebi.ac.uk/jdispatcher/msa/clustalo, accessed on 8 January 2025) [28] within a Singularity container to ensure reproducibility and control over computational resources. Sequences were aligned using the dealign option to eliminate pre-existing gaps, ensuring alignments based solely on sequence data. The software utilized MBED-like clustering for both guide-tree construction and iterative refinements to enhance alignment precision. The output sequences were maintained in their aligned order, and default settings were used for combined iterations, maximum guide tree, and HMM iterations, balancing efficiency, and detailed analysis.

### 2.2. In Silico Analysis of Bovine Classical and Non-Classical MHC Proteins Solubility

SignalP 4.1 (https://services.healthtech.dtu.dk/services/SignalP-5.0/, accessed on 10 January 2025) was used to predict secretion via the classical secretory pathway through the presence of signal peptides; SecretomeP 2.0 Server (https://services.healthtech.dtu.dk/services/SecretomeP-2.0/, accessed on 10 January 2025) was used to predict non-classical secretory pathways. Only the entries that were predicted to be secreted by the SignalP 4.1 or SecretomeP 2.0 Servers were used for further analyses.

The secreted predicted sequences were further analyzed by TMHMM Server v. 2.0 (https://services.healthtech.dtu.dk/service.php?TMHMM-2.0/, accessed on 10 January 2025) for the presence of transmembrane helices. Furthermore, to confirm the secretory nature of the analyzed sequences, two web tools were used to predict the subcellular location of predicted secreted proteins. Meanwhile, TargetP 1.1 Server (https://services.healthtech.dtu.dk/service.php?TargetP-2.0, accessed on 10 January 2025which utilizes neural networks to predict the subcellular location of a given protein sequence, was also used. A similar analysis was performed using WoLF PSORT (http://www.genscript.com/wolf-psort.html, accessed on 10 January 2025), which predicts the protein subcellular location using a dataset of 12,771 animal proteins obtained from UniProt (http://www.uniprot.org/, accessed on 10 January 2025) version 45 annotated by Gene Ontology (http://geneontology.org/, accessed on 10 January 2025).

### 2.3. Placentome Samples

Placentome samples from bovine embryos produced by somatic cell nuclear transfer (SCNT) pregnancies [29] were collected after surgical uteri recovery, and control placentomes were obtained from a local abattoir. For both groups, bovine uteri were incised in the pregnant horn antimesometrial line for placentae assessment. Then, membranes, endometrium, and fetuses were morphologically analyzed. For controls, we only used placentomes derived from pregnancies without gross abnormalities in vasculature, membranes, and placentome number and size. However, for clones, it was known that some placental abnormalities could be present [30,31,32], and only ones without exacerbated abnormalities were used. Placentomes were investigated from clones at early gestation (60 and 90 days; *n* = 5) and near-term (285 ± 0 days; *n* = 6), as well as from controls at early gestation (72.8 ± 10.6 days; *n* = 6) and near term (251 ± 21 days; *n* = 6); these were determined according to their crown–rump length [33]. For RT-qPCR, 16 samples were included, grouped into the following: early gestation (75 ± 17.3 days; *n* = 4) and near term (285 days; *n* = 4) for clones; early gestation (73 ± 16.2 days; *n* = 4) and near term (240 ± 7.4 days; *n* = 4) for controls. The research was approved by the Ethical Committee of the College of Veterinary Medicine and Animal Science of the University of Sao Paulo (protocol number: 9408110315).

### 2.4. Immunohistochemistry Using Qa-2 and IL-A88 Antibodies

Samples were fixed in 4% buffered paraformaldehyde, dehydrated, and paraffin-embedded. Sections of 5 µm were mounted on precoated slides, deparaffinized, and rehydrated. Antigen retrieval was performed by microwaving the slides 4 times in high potency citrate buffer (1.83 mM of monohydrate citric acid and 8.9 mM of sodium citrate tribasic dehydrate, pH 6.0) for 5 min. Endogenous peroxidase was blocked using 3% hydrogen peroxide in distilled water in the dark for 1 h. Nonspecific protein interactions were blocked using 10% of normal goat serum in Tris-buffered saline (TBS: 2.0 mM of Trizma base and 1.36 mM of sodium chloride, pH 7.5) solution for 30 min, followed by incubation with specific primary antibodies Qa-2 (1:50), IL-A88 (1:50) or irrelevant anti-mouse IgG in a humid chamber at 4 °C overnight. The reaction was detected using Dako Advance tm HRP, and the color was developed after applying DAB. The slides were counter-stained with hematoxylin. Between each step, slides were rinsed in TBS 3 times for 5 min, and after antibody incubation, the slides were rinsed in TBS containing 1% normal goat serum. The slides were analyzed using an upright Zeiss Axioplan 2 microscope and Axiocan HRc camera (Zeiss, Jenna, DE).

### 2.5. Detection of MHC Gene Expressions by RT-qPCR

The RNA from all samples was extracted using the RNeasy Plus Mini kit; DNA digestion was performed using amplification grade DNAse I, and cDNA conversion was conducted using the High-Capacity cDNA Reverse Transcription kit, all following the manufacturer’s instructions. For each real-time qPCR reaction, 7.5 μL of SyBr Green PCR master mix, 1.0 mM of each primer (Table 1) [34,35,36,37,38], 1.0 μL of diluted cDNA (63.0 μL of ultrapure water and 1.0 μL of cDNA), and ultrapure water were used to a total volume of 15.0 μL. The default cycle program from the 7500 fast real-time PCR system (Invitrogen) was used. Data were analyzed according to the dCt method from Livak et al. [39], and statistical analysis was performed using least square means analysis of variance with the GLM procedure of SAS (version 5.1). The model included main effects (group and animal) and standard errors. Graphs were plotted on SigmaPlot software (version 12.0).

## 3. Results

### 3.1. Qa-2 and IL-A88 Antibodies Recognize Putative Soluble MHC-I Proteins

**Figure 1:**
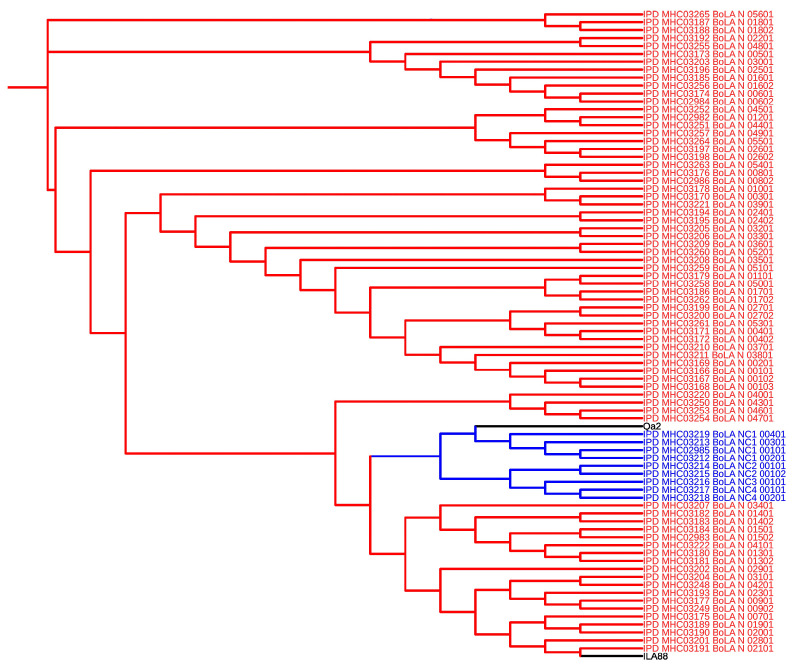
Cluster analysis of MHC I isotype and peptide sequences recognized by Qa-2 (X80933.1) and IL-A88 (NM_001201460.1) antibodies.

The sequence alignment of BoLA isoforms and specific peptides recognized by Qa-2 and IL-A88 antibodies revealed that the Qa-2 antibody predominantly clustered with non-classical bovine MHC I isoforms, while the IL-A88 antibody clustered with classical isoforms, as illustrated in the generated guide tree (Figure 1). In addition in silico prediction of soluble motifs of bovine MHC I isotypes can be accessed in Table S1.

### 3.2. Immunohistochemistry with Distinct Patterns Between Qa-2 and IL-A88 Antibodies

**Figure 2:**
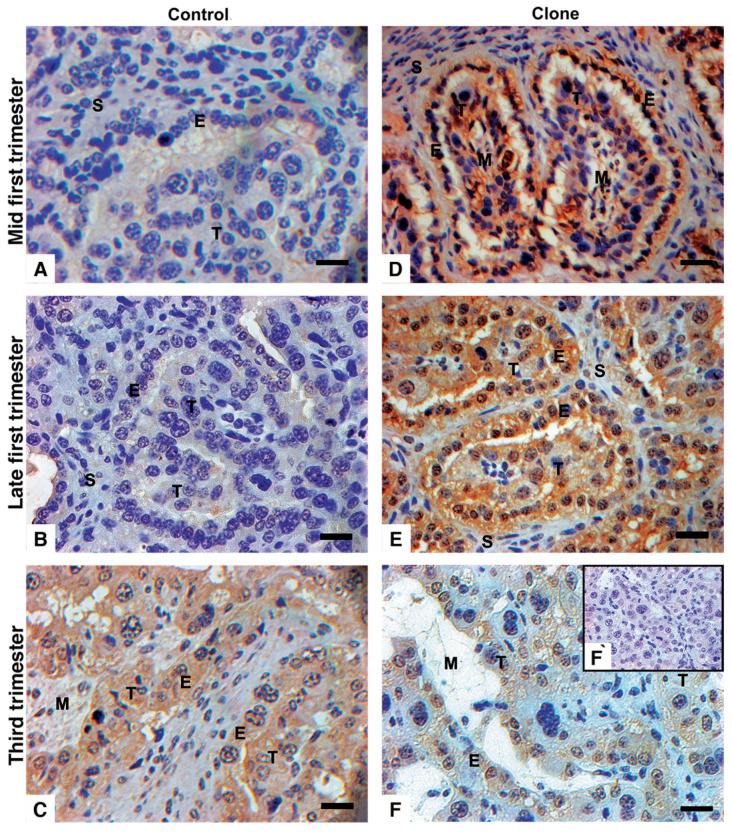
Detection of MHC by Qa-2 antibody in bovine placentomes from control and cloned pregnancies. (**A**) Control at 60 days of pregnancy. Absence of Qa-2 in all tissues. (**B**) Control at 90 days of pregnancy. Absence of Qa-2 expression. (**C**) Control at term. Increased Qa-2 expression restricted to trophoblast and maternal epithelium. (**D**) Clone at 60 days of pregnancy. Qa-2 restricted to trophoblast and maternal epithelium. (**E**) Clone at 90 days of pregnancy. Increased Qa-2 labeling in trophoblast and maternal epithelium and low labeling in trophoblast giant cells near maternal epithelium. (**F**) Clone at term. The same pattern as (**E**) but with low intensity. (**F**`) IgG control. Brown is an immune reaction product; blue is a hematoxylin counterstain. Endometrial stroma (S), maternal epithelium (E), trophoblast (T), and mesenchyme (M). Bar = 20 µm.

**Figure 3:**
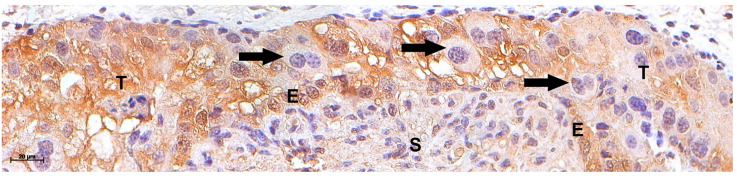
Detection of MHC by Qa-2 antibody in bovine clone placentomes at 90 days of pregnancy. Trophoblastic giant cells (TGCs—arrow) migrate into disorganized maternal epithelium (E). The deeper the TGCs were in the maternal epithelium, the lower the Qa-2 expression. Brown: immune reaction product; blue: hematoxylin counterstain. Endometrial stroma (S), maternal epithelium (E), trophoblast (T), and TGC (arrow). Bar = 20 µm.

In early gestation, controls showed no labeling for the Qa-2 antibody in all tissues of the placentomes (Figure 2A,B). Comparatively, strong staining against Qa-2 antibody stain was observed in near-term uninucleate trophoblasts and the maternal uterine epithelium. In contrast, the bi-nucleated trophoblast was weakly stained, and the mesenchyme and endometrial stroma were largely negative (Figure 2C). In clones, trophoblasts and the maternal epithelial cells were strongly labeled against Qa-2 antibody in early gestation (Figure 2D,E) but only weakly stained near term (Figure 2F). The Qa-2 antibody labeling was stronger in the arcade zones than in the villous areas in all positive cases. In addition, TGCs that were closely apposed to or fused with maternal epithelial cells showed weak to no labeling for Qa-2 (Figure 3).

**Figure 4:**
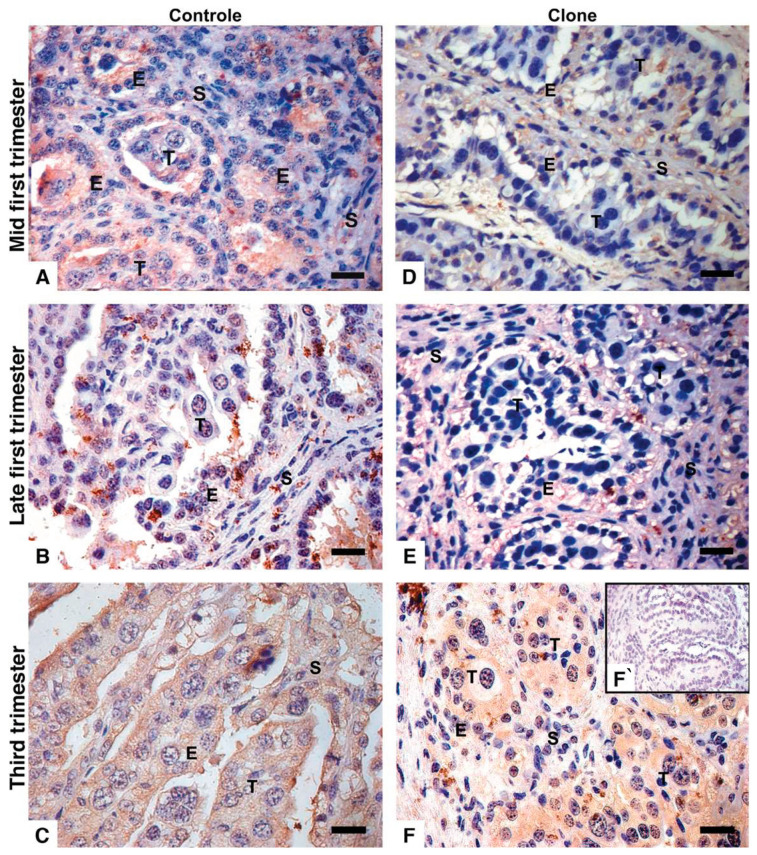
Immunohistochemistry for IL-A88 in bovine placentomes from control and cloned pregnancies. (**A**) Control at 60 days of pregnancy, the anti-IL-A88 signal is observed in the maternal epithelium and light expression in the trophoblast. (**B**) Control at 90 days of pregnancy, anti-IL-A88 is absent in trophoblast. (**C**) Control at term, low presence of anti-IL-A88 in maternal epithelium and trophoblast. (**D**) Clone at 60 days of pregnancy. (**E**) Clone at 90 days of pregnancy. The maternal epithelium has a light IL-A88 signal in both (**D**,**E**), whereas it is absent in trophoblasts. (**F**) Clone at term, the presence of anti-IL-A88 in the maternal epithelium and trophoblast with more intensity than in controls (**C**). Brown is an immune reaction product; blue is a hematoxylin counterstain. (**F`**) IgG control. Endometrial stroma (S), maternal epithelium (E), trophoblast (T), and mesenchyme (M). Bar = 20 µm.

In controls, at 60 days of gestation, both trophoblast and maternal epithelial cells exhibited moderate labeling for IL-A88 antigen (Figure 4A). By day 90 of gestation, while the labeling intensity of IL-A88 in maternal cells remained consistent, it was markedly decreased in the trophoblast cells (Figure 4B). Near-term, all samples displayed faint to weak staining for IL-A88 (Figure 4C). In clone samples, the IL-A88 antibody showed mild to moderate labeling, which was observed only in maternal cells during early gestation (Figure 4D,E). Moreover, in the near term, the IL-A88 antibody labeling intensity was evident in both the trophoblast and maternal epithelium (Figure 4F). Additionally, the TGCs that were closely apposed or fused with the maternal epithelium exhibited markedly weak to no labeling for IL-A88, similar to the staining patterns observed with the Qa-2 antibody. 

### 3.3. Expression and Regulation of MHC I Gene Isoforms

**Figure 5:**
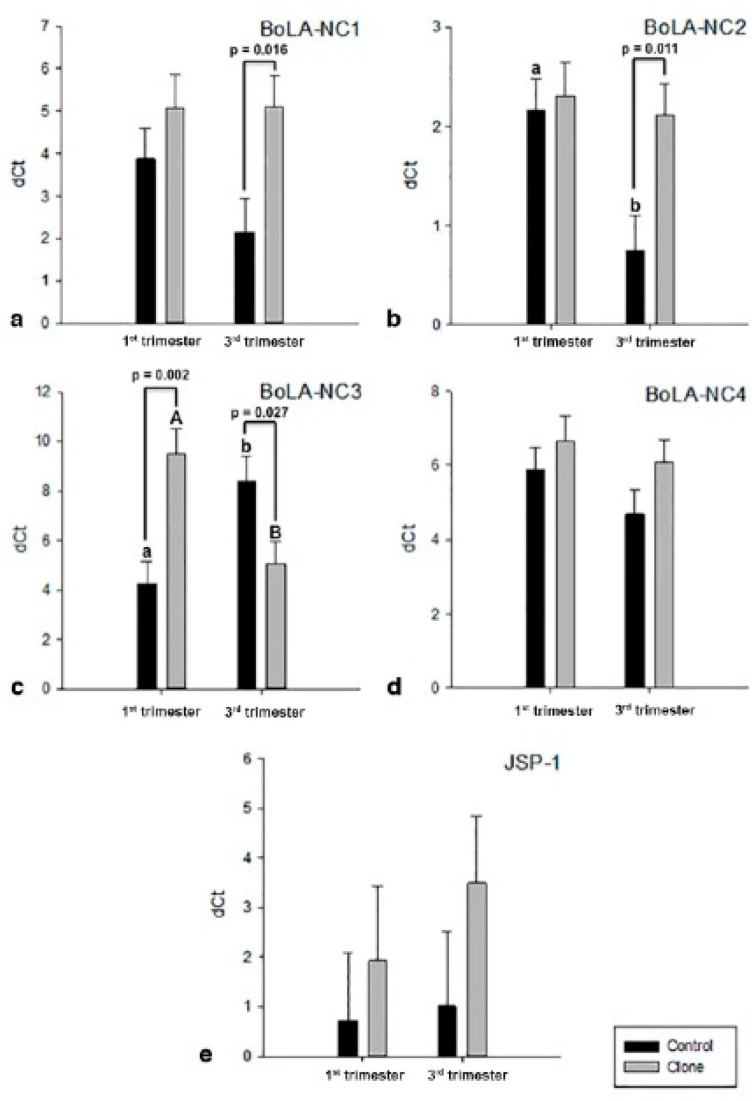
Expression of *BoLA-NC1* (**a**), *BoLA-NC2* (**b**), *BoLA-NC3* (**c**), *BoLA-NC4* (**d**), and *JSP-1* (**e**) in bovine placentomes from control (dark bar) and cloned (grey bar) pregnancies. In the X axis, the pregnancy period (first or third trimester); in the Y axis, gene expression is shown in dCt. Data are least squares means ± S.E.M. Lowercase letters represent significance (*p* ≤ 0.05) between control placentomes. Uppercase letters represent significance (*p* ≤ 0.05) between cloned placentomes. Continuous bars represent significance (*p* ≤ 0.05) inside pregnancy age.

Among the four non-classical MHC I isoforms investigated (Figure 5a–e), *BoLA-NC3* exhibited significant differences in expression throughout pregnancy between the control and cloned groups (*p* < 0.01). In the controls compared to the clones, *BoLA-NC3* expression was upregulated in early gestation (*p* < 0.01) and downregulated near term (*p* = 0.03) (Figure 5c). Expression of *BoLA-NC1* differed only in the near term, showing downregulation in controls relative to clones (*p* < 0.02) (Figure 5a). Similarly, *BoLA-NC2* expression differed only in the near term, being downregulated in controls compared to clones (*p* < 0.02). Additionally, controls exhibited a decrease in *BoLA-NC2* expression near term compared to early gestation (*p* < 0.01), whereas its expression remained constant throughout pregnancy in clones (Figure 5b). In contrast, the expression of *BoLA-NC4* and the classical isoform *JSP-1* did not show significant differences at any analyzed stages of pregnancy or between groups (Figure 5d,e).

## 4. Discussion

In contrast to previously known, MHC-I isoforms were detected in both control and clone bovine placentomes throughout pregnancy at mRNA and protein levels. In our study, two different antibodies for immunohistochemistry were tested, and the antibody–antigen reaction was revealed with a biotin-free polymer-based secondary antibody system. Such second-generation antibody systems are more sensitive and specific [40] than the formerly used biotinylated ones [22,23,26,28,36,37]. We concluded that newer, more sensitive reagents are more suitable for detecting MHC I expression, particularly during early bovine gestation when the MHC class I protein expression is evident in our current work. It is possible that the antigen retrieval and blockage of nonspecific site methods previously required by older reagents may have disrupted the integrity of the antigen–antibody binding process, potentially altering epitope availability, reducing binding affinity, or increasing background signal, thereby affecting assay specificity and sensitivity.

We observed intense labeling in uninucleate trophoblasts compared to TGCs. All the TGCs composing the syncytium with the maternal epithelium exhibited a complete loss of labeling. A similar pattern of heterogeneous MHC-I expression in trophoblasts has been described in humans [41,42]. Only the migratory EVT cells express HLAG to suppress the activation of uterine NK cells and resident macrophages. The EVT interacts with maternal immune cells, hence HLAG expression. In contrast, syncytiotrophoblast near the maternal blood system has only light expression of soluble HLAG and does not express membrane-bound HLAG [43]. Thus, comparable to humans, we suppose that the fused cells at the fetal–maternal interface were also characterized in the bovine by suppressed MHC I expression. This may promote vulnerability regarding the maternal immune system, which may be involved in the process of apoptosis of the syncytia [44] and the release of their products into the maternal system [16]. The strong staining on the maternal side and putative soluble pattern of BoLA isoforms suggest the presence of (a) membrane-bound, classical MHC I as specific for epithelium and (b) soluble isoforms that must have been released by the trophoblast and perhaps “accumulated” in the uterine epithelium [45,46]. In humans, the maternal epithelium displays a similar “accumulation” of non-classical MHC I as observed in the bovine placenta. Even though the bovine placenta is generally less invasive, studies have proven the permeability of fetal products to the maternal system [17,47,48,49]. Recently, the cloned bovine endometrial immune response was shown to be regulated by trophoblast BoLA expression [50] and hypoxic conditions [51], which is a common feature in cloned pregnancies [52,53].

Our results highlight differences between the two antibodies used. In our in silico analysis, the Qa-2 sequence appeared closer to non-classical BoLA isoforms. Moreover, Qa-2 antigens were not detected in early gestation, with markedly increased labeling in the control samples in the near term. In contrast, the human placenta expresses HLA-G from the first trimester onwards [54], probably due to a more invasive placenta. In contrast, clones showed intense labeling in early gestation that decreased close to term, indicating that an altered overexpression of non-classical MHC I during early gestation may lead to pregnancy abnormalities, as evidenced by its expression by chorioallantoic membranes prior to placentome formation [25], which were even higher in MHC-I heterozygous incompatible clones [27]. In our in silico analysis, IL-A88 protein sequences appeared closer to classical MHC I proteins. In control samples, IHC for IL-A88 showed consistent labeling in the maternal epithelium at both early and later stages of gestation. In contrast, clones exhibited weak to no IL-A88 labeling in the maternal epithelium during early gestation but intense labeling in both maternal and fetal epithelium near term. These data suggest that IL-A88 may recognize the characteristics, i.e., classical antigens of epithelia, that become downregulated on the fetal side, similar to those previously observed for humans and mice [55,56]. However, in clones in the near term, the trophoblast is labeled for IL-A88 antigens. The anti-Qa-2 and anti-IL-A88 monoclonal antibodies differed in the pattern of labeling in the control and cloned samples; it could be a consequence of an abnormal chromatin remodeling due to abnormal patterns of methylation of the trophoblast during the preimplantation period that perpetuated during the placental development [57].

*MHC I* mRNA expression was detected in all bovine placentome samples throughout pregnancy, consistent with the isoform evaluations by Santos et al. [58]. Our findings reveal significant variations in the non-classical isoforms, with *BoLA-NC3* being the most regulated gene during pregnancy. The expression patterns of this gene closely correlated with the immunohistochemistry results for Qa-2 antigens observed in this study. The abnormal Qa-2 labeling was especially aligned with non-classical isoform expression in cloned samples. In addition, *BoLA-NC1* and *BoLA-NC2* were significantly upregulated in the near-term stages of clones; however, the functional meaning remained unclear.

## 5. Conclusions

Our data suggest that MHC-I protein and gene expressions in the bovine placenta are similar to those observed in more invasive placenta types, such as in humans and mice, suggesting bovine epitheliochorial placentas possess similar mechanisms of trophoblast evasion from the maternal immune system during gestation. Controls and clones had distinct protein expression profiles for Qa-2 and IL-A88 antigens, which was somehow observed via differences in the expression of non-classical MHC-I molecules. The disparity in MHC-I regulation in clones may lead to a failure in proper pregnancy signaling, potentially leading to gestational failures.

## Figures and Tables

**Table 1:** Details of primers used for expression of classical and non-classical transcripts.

Gene Symbol	Gene Name	Gene ID	Sequence (5′–3′)	Amplicon	Ref.
*BOLA-NC1*	MHC 1 non-classical 1		F: GGATACAAAAGGAAAACACACAGACTT	77	[34]
			R: GGCCTCGCTCTGGTTGTAGT		
*BOLA-NC2*	MHC 1 non-classical 2		F: TGCGCGGCTACTACAACCA	67	
			R: ACGTCGCAGCCGTACATCTC		
*BOLA-NC3*	MHC 1 non-classical 3		F: CCTGCGCGGCTACTACAAC	70	
			R: CACTTCGCAGCCAAACATCA		
*BOLA-NC4*	MHC 1 non-classical 4		F: TTGCGAACGGGCTTGAAC	74	[35]
			R: ACCCACTGGAGGGTGTGAGA		
*JSP-1*	MHC-class I JSP-1	407173	F: TAGGAGTTAGGGAGACTGGCCC	121	[36]
			R: AGCCTCGTCTCTGCTGGAAC		
*YWHAZ*	Tyrosine 3-monooxygenase/tryptophan 5-mono-oxygenase activation protein, zeta	287022	F: GCATCCCACAGACTATTTCC	150	[37]
			R: GCAAAGACAATGACAGACCA		
*ACTB*	Beta-actin	280979	F: CAGCAGATGTGGATCAGCAAGC	108	[38]
			R: AACGCAGCTAACAGTCCGCC		
*PPIA*	Peptidylprolyl isomerase A	281418	F: CATACAGGTCCTGGCATC	91	[38]
			R: CACGTGCTTGCCATCCAA		

## Data Availability

All data are contained within the article and Supplementary Materials.

## Supplementary Materials

The following supporting information can be downloaded at: https://www.mdpi.com/article/10.3390/vetsci12030196/s1, Table S1: In silico prediction of soluble motifs of bovine MHC I isotypes.
