# Supplementary material for: Expression of MHC I Isoforms in Bovine Placentomes: Impact of Cloning
Source: Vet Sci. 2025 Feb 21;12(3):196. doi: 10.3390/vetsci12030196 (PMC11946372; doi:10.3390/vetsci12030196)
Supplement: Supplementary file 1 [file vetsci-12-00196-s001.zip › vetsci-3453138-supplementary.pdf]

Table S1: In silico prediction of soluble motifs of bovine MHC I isotypes

					SignalP-4.1		Targetp v1.1			WoLF PSORT II		Phobius	
**Accession**	**GI#**	**Name**	**Allele**	**Reading Frame**	**D**	**SP**	**SP score**	**Loc**	**RC**	**Loc**	**Score**	**TM**	**SP**
**L02833.1**	**289423**	**MHC class I related mRNA sequence**	3*01001	5'3' Frame 1	0.804	Yes	0.87	S	2	extr	17.0	1	Yes
**L02833.1**	**289423**	**MHC class I related mRNA sequence**	3*01001	3'5' Frame 1	0.804	Yes	0.87	S	2	extr	17.0	1	Yes
**DQ121196.1**	**72536898**	**isolate 19_20+_+B03.1_RC MHC class I antigen (BoLA) gene, partial cds**	3*05201	5'3' Frame 2	0.796	Yes	0.873	S	2	extr	13.5	1	Yes
**DQ121143.1**	**72536792**	**isolate 17_18+_+C04.1 MHC class I antigen (BoLA) gene, partial cds**	1*06101	5'3' Frame 2	0.863	Yes	0.924	S	2	extr	13.0	1	Yes
**BC105263.1**	**75948226**	**MHC Class I JSP.1, mRNA (cDNA clone MGC:129071 IMAGE:8121647), complete cds**	5*07201	5'3' Frame 1	0.796	Yes	0.863	S	2	extr	13.0	1	Yes
**DQ121139.1**	**72536784**	**isolate 15_16+_+D06.1 MHC class I antigen (BoLA) gene, partial cds**	1*01903	5'3' Frame 2	0.855	Yes	0.891	S	2	extr	12.0	1	Yes
**DQ121168.1**	**72536842**	**isolate 15_16+_+A06.1 MHC class I antigen (BoLA) gene, partial cds**	2*04701	5'3' Frame 2	0.795	Yes	0.873	S	2	extr	11.5	1	Yes
**DQ121184.1**	**72536874**	**isolate 15_16+_+F03.1 MHC class I antigen (BoLA) gene, partial cds**	3*01101	5'3' Frame 2	0.798	Yes	0.873	S	2	extr	11.0	1	Yes
**DQ121180.1**	**72536866**	**isolate 15_16+_+E12.1 MHC class I antigen (BoLA) gene, partial cds**	2*04801	5'3' Frame 2	0.795	Yes	0.868	S	2	extr	10.0	1	Yes
**DQ121144.1**	**72536794**	**isolate 17_18+_+A07.1 MHC class I antigen (BoLA) gene, partial cds**	1*04201	5'3' Frame 2	0.841	Yes	0.877	S	2	extr	9.5	1	Yes
**AB259012.1**	**94966481**	**BoLA T2b mRNA for MHC class I antigen, complete cds**	6*04101	5'3' Frame 1	0.809	Yes	0.873	S	2	extr	9.0	1	Yes
**AB259012.1**	**94966481**	**BoLA T2b mRNA for MHC class I antigen, complete cds**	6*04101	3'5' Frame 1	0.809	Yes	0.873	S	2	extr	9.0	1	Yes
**AJ010862.2**	**63092090**	**MHC class I 3349.1 gene**	6*01501	5'3' Frame 1	0.794	Yes	0.878	S	2	extr	9.0	1	Yes
**X80933.1**	**1657247**	**mRNA for MHC class 1 (clone 1)**	.	5'3' Frame 1	0.861	Yes	0.824	S	3	extr	21	0	Yes
**L02832.1**	**289422**	**MHC class I related mRNA sequence**	3*00402	5'3' Frame 1	0.799	Yes	0.765	S	3	extr	16.0	1	Yes
**L02832.1**	**289422**	**MHC class I related mRNA sequence**	3*00402	3'5' Frame 1	0.799	Yes	0.765	S	3	extr	16.0	1	Yes

**DQ121176.1**	**72536858**	**isolate 15_16+_+D09.1 MHC class I antigen (BoLA) gene, partial cds**	3*02702	5'3' Frame 2	0.795	Yes	0.796	S	3	extr	15.0	1	Yes
**DQ121193.1**	**72536892**	**isolate 15_16+_+A11.1 MHC class I antigen (BoLA) gene, partial cds**	3*05101	5'3' Frame 2	0.794	Yes	0.828	S	3	extr	15.0	1	Yes
**DQ121165.1**	**72536836**	**isolate 19_20+_+G10.1 MHC class I antigen (BoLA) gene, partial cds**	2*04301	5'3' Frame 2	0.794	Yes	0.828	S	3	extr	13.0	1	Yes
**DQ121175.1**	**72536856**	**isolate 15_16+_+B09.1_RC MHC class I antigen (BoLA) gene, partial cds**	3*05301	5'3' Frame 2	0.79	Yes	0.77	S	3	extr	13.0	1	Yes
**DQ121183.1**	**72536872**	**isolate 17_18+_+E07.1 MHC class I antigen (BoLA) gene, partial cds**	4*02401	3'5' Frame 2	0.787	Yes	0.855	S	3	extr	13.0	1	Yes
**Y09205.1**	**1665858**	**MHC class 1 protein molecule D18.1**	4*02401	5'3' Frame 1	0.787	Yes	0.855	S	3	extr	13.0	1	Yes
**DQ121190.1**	**72536886**	**isolate 21_22+_+C10.1_ MHC class I antigen (BoLA) gene, partial cds**	3*05001	5'3' Frame 2	0.799	Yes	0.813	S	3	extr	12.0	1	Yes
**DQ121170.1**	**72536846**	**isolate 17_18+_+H09.1 MHC class I antigen (BoLA) gene, partial cds**	2*04601	5'3' Frame 2	0.794	Yes	0.828	S	3	extr	12.0	1	Yes
**DQ304656.1**	**83583570**	**MHC class I antigen (BoLA-N) mRNA, BoLA-N*01502 allele, partial cds**	6*01502	5'3' Frame 2	0.465	Yes	0.215	S	3	extr	11	1	Yes
**DQ121150.1**	**72536806**	**isolate 17_18+_+A10.1 MHC class I antigen (BoLA) gene, partial cds**	1*02301	5'3' Frame 2	0.85	Yes	0.853	S	3	extr	10.5	1	Yes
**DQ121150.1**	**72536806**	**isolate 17_18+_+A10.1 MHC class I antigen (BoLA) gene, partial cds**	1*02301	3'5' Frame 2	0.85	Yes	0.853	S	3	extr	10.5	1	Yes
**Y09208.1**	**1665864**	**MHC class 1 protein molecule D18.4**	1*02301	5'3' Frame 1	0.85	Yes	0.853	S	3	extr	10.5	1	Yes
**DQ121149.1**	**72536804**	**isolate 15_16+_+G04.1 MHC class I antigen (BoLA) gene, partial cds**	1*00902	5'3' Frame 2	0.85	Yes	0.854	S	3	extr	10.0	1	Yes
**DQ121149.1**	**72536804**	**isolate 15_16+_+G04.1 MHC class I antigen (BoLA) gene, partial cds**	1*00902	3'5' Frame 2	0.85	Yes	0.854	S	3	extr	10.0	1	Yes
**DQ121191.1**	**72536888**	**isolate 17_18+_+C03.1 MHC class I antigen (BoLA) gene, partial cds**	3*01701	5'3' Frame 2	0.797	Yes	0.824	S	3	extr	10.0	1	Yes
**L02835.1**	**289425**	**MHC class I related mRNA sequence**	2*00802	5'3' Frame 1	0.512	Yes	0.502	S	4	extr	10.5	1	Yes
**L02835.1**	**289425**	**MHC class I related mRNA sequence**	2*00802	3'5' Frame 1	0.512	Yes	0.502	S	4	extr	10.5	1	Yes
**DQ121161.1**	**72536828**	**isolate 19_20+_+G12.1 MHC class I antigen (BoLA) gene, partial cds**	2*04401	5'3' Frame 2	0.901	Yes	0.946	S	1	E.R.	12.5	1	Yes
**AJ010865.1**	**3688218**	**MHC class I 4221.1 gene**	1*01901	5'3' Frame 1	0.855	Yes	0.859	S	2	E.R.	10.5	1	Yes
**DQ121177.1**	**72536860**	**isolate 15_16+_+A02.1 MHC class I antigen (BoLA) gene, partial cds**	2*04501	5'3' Frame 2	0.83	Yes	0.93	S	1	E.R.	11.5	1	Yes
**DQ121204.1**	**72536914**	**isolate 25_26+_+E07.1 MHC class I antigen (BoLA) gene, partial cds**	1*04901	5'3' Frame 2	0.83	Yes	0.924	S	1	E.R.	10.5	1	Yes

**AB245424.1**	**84095082**	**BoLA T2a mRNA for MHC class I antigen, complete cds**	2*01201	5'3' Frame 1	0.829	Yes	0.908	S	1	E.R.	9.5	1	Yes
**AJ010867.1**	**3688222**	**MHC class I D18.5 gene**	2*02501	5'3' Frame 1	0.827	Yes	0.886	S	1	E.R.	9.5	1	Yes
**AJ010863.2**	**63092092**	**MHC class I Man1 gene**	2*00801	5'3' Frame 1	0.826	Yes	0.919	S	1	E.R.	10.5	1	Yes
**NM_001201460.1**	**319803124**	**Mus musculus histocompatibility 2, Q region locus 9 (H2-Q9), mRNA**	.	.	0.822	Yes	0.915	S	1	E.R.	16.0	1	Yes
**AJ010861.1**	**3688210**	**MHC class I Man2 gene**	6*01401	5'3' Frame 1	0.817	Yes	0.869	S	2	E.R.	11.0	1	Yes
**BC151402.1**	**154425689**	**MHC Class I JSP.1, mRNA (cDNA clone MGC:179240 IMAGE:7944171), complete cds**	5*06401	5'3' Frame 2	0.797	Yes	0.817	S	3	E.R.	8.5	.	Yes
**AJ010866.3**	**74381873**	**MHC class I Man8 gene**	2*01601	5'3' Frame 1	0.768	Yes	0.888	S	1	E.R.	11.5	1	Yes
**DQ121206.1**	**72536918**	**isolate 21_22+_+B03.1_ MHC class I antigen (BoLA) gene, partial cds**	2*01602	5'3' Frame 2	0.768	Yes	0.888	S	1	E.R.	11.5	1	Yes
**X80934.1**	**1657249**	**mRNA for MHC class 1 (clone 6)**	6*01301	5'3' Frame 1	0.759	Yes	0.882	S	2	E.R.	18.0	1	Yes
**X80934.1**	**1657249**	**mRNA for MHC class 1 (clone 6)**	6*01301	3'5' Frame 2	0.759	Yes	0.882	S	2	E.R.	18.0	1	Yes
**Y09206.2**	**63093868**	**MHC class 1 protein molecule D18.2**	3*01701	5'3' Frame 1	0.797	Yes	0.829	S	3	E.R._golg	10.0	.	Yes
**AJ010864.1**	**3688216**	**MHC class I Man3 gene**	1*02001	5'3' Frame 1	0.863	Yes	0.863	S	2	golg	10.5	1	Yes
**Y09207.1**	**1665862**	**MHC class 1 protein molecule D18.3**	2*01801	5'3' Frame 1	0.725	Yes	0.762	S	4	pla	6.5	1	Yes
